# A curious case of unsuspected intracranial metallic splinter with complications

**DOI:** 10.6026/973206300214031

**Published:** 2025-11-15

**Authors:** Sucheta Tirpude, Dhirav Thakkar

**Affiliations:** 1 Department of Neurosurgery, H.B.T. Medical College and Dr. R.N. Cooper Hospital, Mumbai, India

**Keywords:** Metallic foreign body, intracranial foreign body, ventriculoperitoneal shunt, pseudocyst formation

## Abstract

Penetrating injuries to the brain, due to low-velocity, non-missile objects are rare in civilian lament, but can carry significant morbidity. We describe a 20-year-old male with altered sensorium, headache and imbalance, months following what was deemed a trivial head injury from cricket. After imaging, a 2.5 cm metallic splinter lodged in the left cerebellum, which was subsequently surgically removed. The patient developed meningitis and hydrocephalus, required a shunt thereafter, but was complicated by the development of a pseudocyst, cherry-picking a CSF leak, malfunction of the original shunt and he ultimately had to be managed with a ventriculoarterial shunt. Thus, the need for routine imaging, expedited clinical recognition and individual management of delayed complications for penetrating injuries with the brain.

## Background:

Penetrating brain injuries (PBI) from non-missile, low-velocity objects are highly uncommon in civilian setting, but can yield significant morbidity and mortality. These injuries, unlike high-velocity missile injuries commonly seen in combat, typically occur from everyday objects such as nails, slivers, keys and rods and, while high-velocity missile injuries will generally have an identifiable wound of entry, PBI from civilian mechanisms usually leave what looks like a trivial wound of entry [1]. As a result, these injuries are often unrecognized and unidentified as the small external wound was often considered unimportant by healthcare professionals [2]. As such, the delay in presentation to medical attention creates opportunities for complications (i.e. headache, seizures and meningitis-type features). This problem is further complicated by contamination of the wound tract from bone and/or hair and/or skin and/or and other environment debris which increase the risk of intracranial infection. Standard neuroimaging is critical for the prompt diagnosis of swallowed foreign bodies [3]. Basic x-rays and CT imaging are easily accessible, inexpensive and relatively accurate for identifying metallic or radiopaque pieces (use of MRI should be avoided if a foreign body is suspected because of possible displacement or injury to surrounding tissue) [4]. Tissue involvement in the posterior fossa and cerebellum may be especially troublesome as the limits of working space in this compartment can lead to swift neurological decline caused by mass effect, edema and obstructive hydrocephalus [5]. Moreover, while post-traumatic hydrocephalus is uncommon in civilian PBIs, they may still present acutely, or delayed and may require CSF diversion (e.g., ventriculoperitoneal shunting). PBI management needs a comprehensive process that includes foreign body removal, if possible, infection management and careful monitoring for subsequent complications during the postoperative phase [6]. Complications related to the shunt, such as pseudocyst formation, cerebrospinal fluid leakage, or malfunction can complicate the clinical course necessitating further revision surgery. Because of the infrequency of such cases in civilian practice, the literature is limited and these individual case reports can be informative based on the diagnostic dilemma, complication potential and evolving management styles [7]. Therefore, it is of interest to describe a case of penetrating brain injury caused by a trivial head trauma that resulted in delayed neurological manifestations and complex postoperative complications.

## Case report:

A 20year young male reported to the emergency with history of altered sensorium, headache and lethargy associated with vomiting and accompanied with imbalance in gait. On examination he had neck stiffness with positive Kernig's sign, with pain at the back of head (left occipital area). He was suspected to have tubercular meningitis after a guarded lumber puncture and was planned for MRI -Brain with contrast. The procedure was stalled twice as metal body was suspected on the patient. On repeated questioning, there was no history to support the presence of metallic foreign body. A screening Xray skull showed-a metallic foreign body through the left occipital bone lodged in left cerebellar hemisphere. On further questioning, he gave history of fall while playing cricket 2-3 months back for which he had a check-up with the local physician and the puncture wound was cleaned and dressed. An immediate removal was planned and a small defect in the bone was identified. A small burr hole was made and the foreign body was delivered whole. The foreign body was a rusted metal splinter 2.5 cm in length. MRI Brain showed communicating hydrocephalus. His sensorium became further altered pupillary reaction sluggish for which an immediate ventriculoperitoneal shunt was planned. He tolerated the procedure well and showed a speedy recovery. After 4-6 weeks, he started showing abdominal distension with increasing frequency of headache and nausea. On examination it was evident that there was an abdominal swelling, gradually increasing and partially draining shunt. There was no associated fever or any signs of infection along the shunt. USG-abdomen was suggestive of pseudocyst with shunt tip enclosed in the cyst. Pseudocyst drainage with placement of shunt tip in right paracolic gutter into the pelvis was done with help of general surgeons. He improved, but after 1 week, he again had CSF leak through the abdominal incision. He still was having persistent headache and blurring of vision which progressed to drop in consciousness with papilledema. A ventriculoarterial shunt was planned and carried out and he improved radically. After 2 uneventful years he again had complaints of headache associated with vomiting and MRI Brain showed moderate to gross ventriculomegaly. Subsequently he became increasingly drowsy. Chamber was not compressible, repeated attempts were made to compress chamber, relatives were asked to press the chamber and he was planned for shunt revision the next morning. That evening, patient started improving and the chamber was found to be again compressible. The next morning remarkable improvement in level of consciousness was seen and decision for reversal of shunt was abandoned. Subsequent MRI Brain showed significant decrease in size of ventricles and patient was discharged.

## Discussion:

While the cranium may provide a barrier to penetration, the literature has demonstrated otherwise with accounts of virtually all types of foreign bodies. In the past, most penetrating intracranial foreign bodies were the result of war and gunshot injuries, but other sources also exist. Penetrating intracranial foreign bodies can be the result of civilian firearm incidents, as well as criminal assault, work-related and accident-related injuries (e.g., nails, wood and shrapnel) and equipment [8]. Falls and road traffic injuries have also contributed to penetrating injuries where the source was the driver or passenger incidentally. Rarely, some patients with psychiatric illness have been found to have foreign insertions due to self-infliction [9]. There have even been reports of infanticide utilizing a sewing needle where almost a third occur incidental to a more serious injury. A classic review by Lichtenstein in 1936 outlined a whole bunch of preferential offending objects including knives, crocheting needles, pitchfork prongs, knitting needles, nails, wood and keys [10]. This case is atypical in that the intracranial foreign body was not suspected and presented as meningitis months later after a trivial cricket injury. The patient first sought care but was reassured the puncture wound was minimal and not imaging was performed, reflecting the deceptiveness of puncture wounds, as noted previously [11]. Our patient went on to have meningitis with subsequent hydrocephalus that required ventriculoperitoneal shunting and the injury mechanism was not identified until much later. The infratentorial position of the foreign body is particularly concerning due to the increased risk of hydrocephalus and rapid change in clinical condition [12]. CT imaging and plain radiography is easy to obtain, inexpensive and should be considered part of the assessment of any head wound with concern for penetration [13, 14]. However, injuries in resource-poor or austere settings may never undergo imaging and, even in the absence of a clear external wound, first responders may not evaluate a patient for injury [4]. Consequently, foreign bodies within the cranium can go undiagnosed until the patient presents for infection, seizures, or chronic headaches [15]. In our case, we were unable to obtain repeat MRIs due to metallic artifact. This reinforced the notion that MRI is contraindicated when there are concerns for metallic foreign bodies due to the possibility of displacement and potential injury to tissues. A screening CT, which ultimately localized the metallic sliver, allowed for surgical removal of the foreign body [16]. There was a high potential for infection after all penetrating brain injury due to the introduction of pathogen bacteria into the wound tract through skin, hair, bone and dirt contamination. Meningitis, ventriculitis and abscess presentations have been reported for both combat and civilian populations [17]. The civilian presentation of nail-gun injury is probably lower in energy transfer, but may still result in meningitis; and their overall infection rate appears to be less than craniocerebral gunshot injury. Our patient developed meningitis approximately 2-3 months after their injury in combination with military reports that the majority of infections after penetrating brain injuries occur within 6-8 weeks although a delayed presentation of infection has been described [18]. Survivability is commonly associated with the location of the injury; penetrating trauma to the frontal lobes is more likely to have a positive outcome than other types of penetrating damage. Deep structures such as the cerebellum like in this case are less commonly involved and pose significantly greater risks [19]. Almost all civilian reports pertaining to unsuspected foreign bodies in the posterior fossa, occurred months later with meningitis or hydrocephalus. These reports demonstrate the key of how deceptive trivial trauma may seem, while reinforcing the need for rapid radiological assessment and case specific management. The case underlying our patient demonstrates that a successful shunt placement does not end the clinical challenges, as shunt-related complications may occur (e.g., pseudocyst formation, CSF leakage and transient malfunction) that necessitate further interventions. Overall, this case and previously published reports, highlights the need to carefully follow up with the patient, make accurate diagnoses of intracranial foreign bodies and tailor treatment plans to the region of the body it is located in, the timing of presentation and the local complications.

## Conclusion:

Brain injuries from low-velocity penetration can take on delayed or misleading appearances, underscoring the need for imaging in the acute phase of symptoms. Management will be individualized to the specific case, with emphasis on monitoring potential complications of surgical management (for example: infection and shunt failure). Thus, we show the vigilance needed in diagnosing and adequately addressing low-velocity penetrating brain injuries.

## Disclosure statement: 

The authors report there are no competing interests to declare.

## Consent:

This is to certify that we, the authors, have obtained written and informed consent from the participant.
